# Structure of the 5′ Untranslated Region of Enteroviral Genomic RNA

**DOI:** 10.1128/JVI.01288-19

**Published:** 2019-11-13

**Authors:** Bejan Mahmud, Christopher M. Horn, William E. Tapprich

**Affiliations:** a Biology Department, University of Nebraska at Omaha, Omaha, Nebraska, USA; Instituto de Biotecnologia/UNAM

**Keywords:** 5' untranslated region, coxsackievirus B3, RNA structure, internal ribosome entry site

## Abstract

Enteroviral RNA genomes share a long, highly structured 5′ untranslated region (5′ UTR) containing a type I internal ribosome entry site (IRES). The 5′ UTR is composed of stably folded RNA domains connected by unstructured RNA regions. Proper folding and functioning of the 5′ UTR underlies the efficiency of viral replication and also determines viral virulence. We have characterized the structure of 5′ UTR genomic RNA from coxsackievirus B3 using selective 2′-hydroxyl acylation analyzed by primer extension (SHAPE) and base-specific chemical probes in solution. Our results revealed novel structural features, including realignment of major domains, newly identified long-range interactions, and an intrinsically disordered connecting region. Together, these newly identified features contribute to a model for enteroviral 5′ UTRs with type I IRES elements that links structure to function during the hierarchical processes directed by genomic RNA during viral infection.

**IMPORTANCE** Enterovirus infections are responsible for human diseases, including myocarditis, pancreatitis, acute flaccid paralysis, and poliomyelitis. The virulence of these viruses depends on efficient recognition of the RNA genome by a large family of host proteins and protein synthesis factors, which in turn relies on the three-dimensional folding of the first 750 nucleotides of the molecule. Structural information about this region of the genome, called the 5′ untranslated region (5′ UTR), is needed to assist in the process of vaccine and antiviral development. This work presents a model for the structure of the enteroviral 5′ UTR. The model includes an RNA element called an intrinsically disordered RNA region (IDRR). Intrinsically disordered proteins (IDPs) are well known, but correlates in RNA have not been proposed. The proposed IDRR is a 20-nucleotide region, long known for its functional importance, where structural flexibility helps explain recognition by factors controlling multiple functional states.

## Abstract (precis)

Enterovirus infections are responsible for human diseases, including myocarditis, pancreatitis, acute flaccid paralysis, and poliomyelitis. The virulence of these viruses depends on efficient recognition of the RNA genome by a large family of host proteins and protein synthesis factors, which in turn relies on the three-dimensional folding of the first 750 nucleotides of the molecule. Structural information about this region of the genome, called the 5′ untranslated region (5′ UTR), is needed to assist in the process of vaccine and antiviral development. This work presents a model for the structure of the enteroviral 5′ UTR. The model includes an RNA element called an intrinsically disordered RNA region (IDRR). Intrinsically disordered proteins (IDPs) are well known, but correlates in RNA have not been proposed. The proposed IDRR is a 20-nucleotide region, long known for its functional importance, where structural flexibility helps explain recognition by factors controlling multiple functional states.

## INTRODUCTION

The genus *Enterovirus* includes 15 species of animal viruses that belong to the family *Picornaviridae* (1). Each species contains many subtypes and variants, with over 80,000 enteroviral genome sequences available in the nucleotide database. The diversity of the viruses in the genus is reflected by the diversity of disease processes that result from infection. In humans, seven species of enteroviruses are responsible for disease states that range from mild conditions like the common cold to very severe diseases, such as poliomyelitis, myocarditis, and acute flaccid paralysis (2). Despite the broad diversity of virus types and diseases, enteroviruses are remarkably similar in structural organization and infection mechanisms. They are small, nonenveloped, icosahedral viruses with a positive (+)-polarity single-stranded RNA genome typical of the *Picornaviridae* (3, 4). Enteroviruses also share a highly structured 5′ untranslated region (5′ UTR) of approximately 750 nucleotides with a conserved domain organization and a type I internal ribosome entry site (IRES) (5–7). The enteroviral 5′ UTR is responsible for recruiting ribosomes, canonical initiation factors, and noncanonical host factors, collectively known as IRES *trans*-acting factors (ITAFs), to initiate translation of the viral polyprotein via the IRES element (8) and also provides an RNA domain called the cloverleaf on the 5′ end that is recognized by ITAFs and the viral polymerase for genome replication (9). We have characterized the RNA structure in the 5′ UTR of coxsackievirus B3 (CV-B3) as a model of enteroviral genomic RNA.

CV-B3 is an important human pathogen. While most infections are inapparent, they can lead to serious and even fatal diseases, such as pancreatitis, myocarditis, and dilated cardiomyopathy (10–15). The 5′ UTR of CV-B3 and the closely related poliovirus (PV) has been studied in detail because it participates in all parts of the infection cycle and contains critical virulence determinants (16–20). During infection, the 5′ UTR orchestrates a hierarchical series of activities that ensure efficient gene expression and replication. In general, translation and replication are controlled by different 5′ UTR RNA domains. The highly conserved cloverleaf structure (domain I) directs replication of both positive and negative strands (9, 21–25). Domains II to V work together to form the viral IRES that controls translation. In enteroviruses, the IRES is classified as type I and is distinct from the type II and III, HCV-like, and IGR IRES elements present in other picornavirus genera (26).

As genome sequences became available and solution techniques for determining RNA structure were developed, models of the enteroviral 5′ UTR were proposed (27–29). These models have been refined as experimental systems have advanced and as more sequences have become available for detailed comparative analyses (6, 20). Significant advances in methods that interrogate RNA structure in solution, most notably selective 2′-hydroxyl acylation analyzed by primer extension (SHAPE) (30, 31), together with highly advanced RNA-folding algorithms that incorporate SHAPE constraints into thermodynamic and comparative structure predictions (32), make it possible to propose significant changes to the existing models. We have applied these new approaches and present a model for the 5′ UTR of CV-B3 that adds structural information in three major ways. First, although the domain structure of previous models is recognizable and domains I and IV have only minor adjustments, there is significant reorganization of domains II, III, V, and VI. Second, our analysis has identified two new long-range interactions, one that has base pairing that is conserved in enteroviral 5′ UTRs and another that has base pairs that are CV-B3 specific. Third, we propose that the conserved pyrimidine-rich region downstream of domain V with the motif Y*_n_*-X*_m_*-AUG (33, 34), where Y*_n_* is 8 to 10 pyrimidines, X*_m_* is 18 to 20 nucleotides, and AUG is a cryptic start codon, is an intrinsically disordered RNA region (IDRR). The Y*_n_*-X*_m_*-AUG region is known to be important for binding multiple factors, including the ribosome (35). Similar to the role of intrinsically disordered regions in proteins (36), this disordered RNA region may facilitate multiple hierarchical interactions.

## RESULTS

### 5′ UTR secondary-structure model.

We combined data from three complementary approaches to propose a new model for the secondary structure of the 5′ UTR of enteroviral genomic RNA: chemical modification in solution, multiple-sequence analysis, and free-energy analysis using maximum expected accuracy (MEA) calculations. These three approaches were applied to the 5′ UTR of CV-B3 as a model for enteroviruses. The results from the three types of analysis provided inputs for constraining base-pairing interactions in the TurboFold II folding algorithm (32).

Data from chemical modification in solution was generated by SHAPE and was supplemented by the base-specific chemical probes dimethyl sulfate (DMS) and 1-cyclohexyl-(2-morpholinoethyl) carbodiimide metho-*p*-toluene sulfonate (CMCT). SHAPE chemicals, such as *N*-methylisatoic anhydride (NMIA), target and react with the 2′-OH group of the RNA sugar backbone. The reactivity of an individual base depends on the flexibility of its ribose backbone, which is determined by base pairing and other conformational constraints. More flexible positions are modified to a greater extent. For further structural analysis, we utilized two base-specific chemical probes that helped to distinguish between positions involved in Watson-Crick pairs and positions that are unpaired. For both SHAPE and base-specific probing, modified sites were identified by reverse-transcriptase (RT) extension of fluorescently labeled cDNA primers. The cDNA fragments were separated using capillary electrophoresis, and the resulting fluorescence intensity files were analyzed using ShapeFinder (37). Modification intensities from ShapeFinder were inputs for TurboFold II, a structural prediction algorithm that relies on experimentally generated probing data and sequence conservation among functionally homologous sequences. For the sequence data set, we selected all of the 5′ UTR sequences available in the NCBI database where published characterization indicated that the genomes were derived from virulent, wild-type strains of CV-B3 (see Table S1 in the supplemental material).

**FIG 1:**
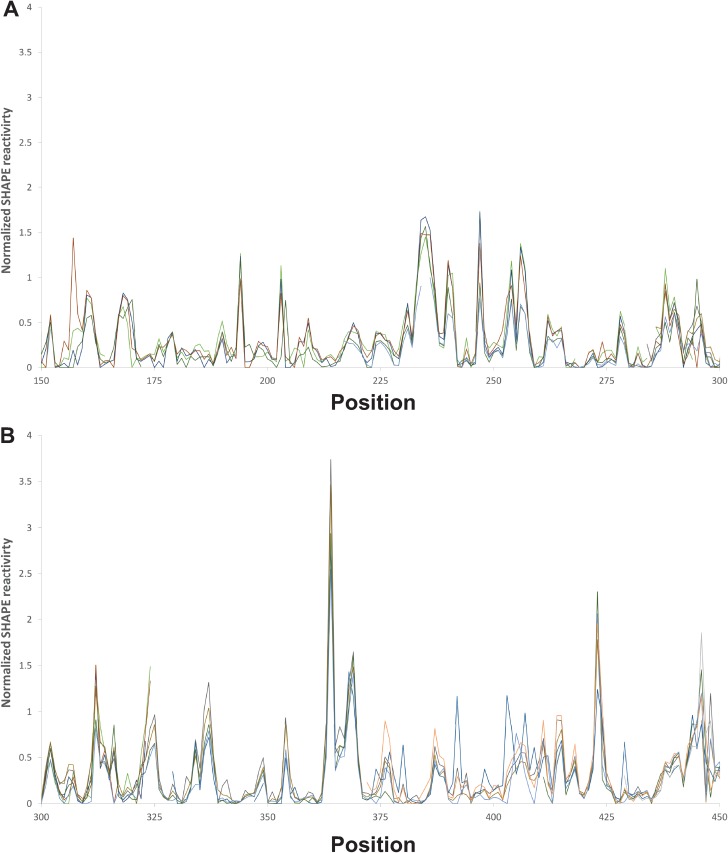
SHAPE reactivity traces by position. Reactivity traces for independent SHAPE experiments are overlaid on the graphs. Raw data for all SHAPE reactivity values for all experiments and all positions, together with means and standard deviations for each position, are listed in Table S3. (A) Reactivity traces for positions 150 to 300. (B) Reactivity traces for positions 301 to 450.

**FIG 2:**
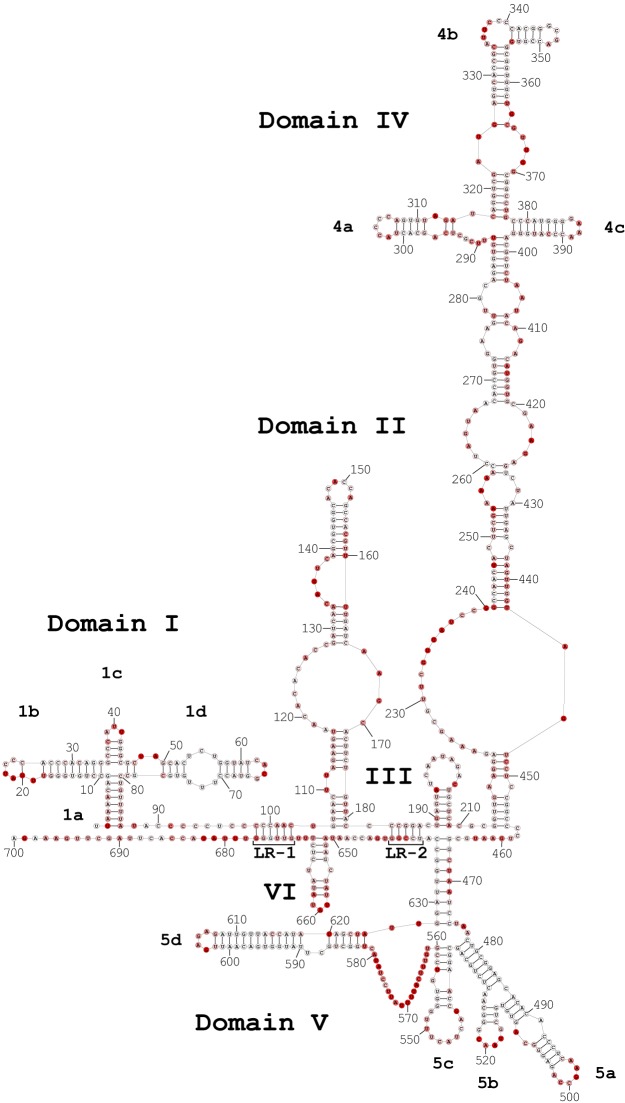
Secondary-structure model for the CV-B3 5′ UTR. SHAPE reactivity at each position is indicated by red shading. No shading shows no/low reactivity, with a normalized reactivity value below 0.3. Light-red shading shows moderate reactivity, with a normalized reactivity value between 0.3 and 0.7. Dark-red shading shows high reactivity, with a normalized reactivity value above 0.7. Domains are labeled I to VI. Stem-loops in domains with junction loops are labeled with letters. Two long-range interactions are labeled as LR-1 and LR-2.

**FIG 3:**
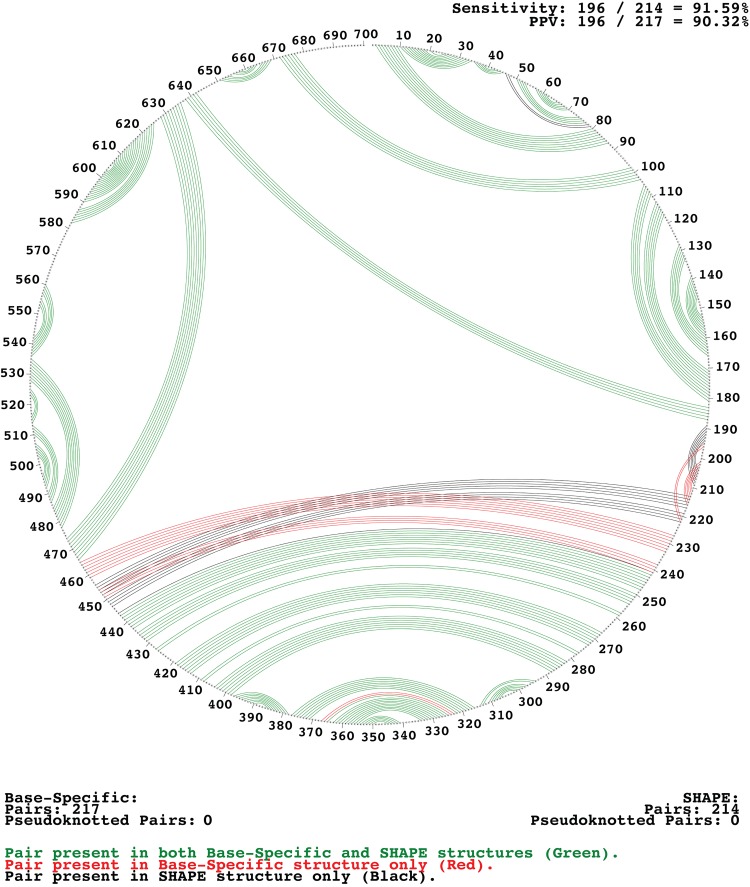
Circle diagram comparing secondary-structure models generated by SHAPE reactivity and base-specific reactivity. Nucleotide positions are displayed outside the circle. Base pairs are connected by lines inside the circle. Differences in the models generated are shown in red and black. PPV, positive predictive value.

SHAPE data (Fig. 1; see Table S2 in the supplemental material) were used to generate a secondary structure of the CV-B3 5′ UTR (Fig. 2). Base-specific probes, DMS and CMCT, were used in parallel to confirm the findings of SHAPE probing. When the base-specific data set was used independently in the TurboFold II analysis, the overall resultant structure prediction was over 92% similar to the one generated with SHAPE alone (Fig. 3). Comparison of the two predictions showed that the overall structure of each domain was preserved, including the newly proposed long-range interactions. The differences were primarily small extensions or reductions of base-paired stem regions. These differences included small changes in domains I, III, and IV. In domain I, the base-specific data set removed 2 base pairs that increase the size of a loop region. In domain III, the basal stem was extended, which shortened the basal stem of domain IV. The interconnecting region between domains IV and V was also shortened.

Two models of the CV-B3 5′ UTR structure proposed previously (6, 38) identified 11 (A to K) and 7 (I to VII) domains defined by base-paired basal stems. Our new model maintains some domains from both models but makes substantial changes to the structural organization of the majority of the molecule. Our model confirms the structure of domain I, also called the cloverleaf. Extensive characterization of the cloverleaf showed that the domain plays a critical role in initiation of replication by forming ribonucleoprotein (RNP) complexes with both viral and host factors (24, 25, 39). A single G-C pair (positions 20 and 24) is proposed to close the apical part of stem-loop b in domain I. The high SHAPE reactivity of 20G indicates that this pair may not be stably formed, in which case the apical part of stem-loop b would consist of a single larger loop that includes the nucleotides within the base pair and the bulge (19U, 20G, and 24C). This matches the proposed model of stem-loop b of the human rhinovirus 14 cloverleaf as determined by nuclear magnetic resonance (NMR) (40).

The interconnecting region between domains I and II has been shown to interact with poly(rC) binding protein 2 (PCBP2) with high affinity (41). Position 91C of the C-rich region near the 3′ end of the cloverleaf (positions 90 to 94) is moderately reactive; however, only 92C and 93C are highly conserved (97%). Overall, the pyrimidine-rich region between domains I and II stretches between positions 88 and 104 and is mostly protected from modification.

Domain II has been suggested to be crucial for viral virulence (17, 18, 20, 42, 43). In our model, domain II occupies the region between positions 105 and 181, which is larger than the model previously reported by Bailey and Tapprich for CV-B3 (6), as well as a recently proposed model for poliovirus (44), but identical to the model proposed by Liu et al. (38) and the model proposed by Zell and Stelzner for bovine enterovirus (28).

The predicted secondary structure of domain III consists of a single stem-loop occupying the region between bases 189 and 209, much shorter than the domain previously reported by Bailey and Tapprich, which included multiple stems and internal loops (6), but consistent with the small domain proposed by Liu et al. (38). Interestingly, deletion of the nucleotides included in the shorter domain does not inhibit poliovirus replication and translation (45–47).

As consistently documented in previous models, the >200-nucleotide domain IV assumes a very complex structure. The majority of our proposed model for domain IV is consistent with prior models, particularly near the apex of the complex stem-loop domain. The junction loop atop domain IV branches out into a cruciform structure consisting of stem loops 4a, 4b, and 4c. Both stem-loops 4a and 4b have single-stranded poly(rC) regions, which may be involved in binding to poly(rC) binding proteins. The poly(rC) region within the apical loop of stem-loop 4a (positions 303 to 306) is highly conserved among enteroviruses but was not reactive when tested with SHAPE; it did, however, exhibit moderate reactivity when probed with base-specific agents. The poly(rC) region within stem-loop 4b (positions 338 to 341) is also highly conserved across the enterovirus A, B, and C sequences and is largely inaccessible to SHAPE modification. While position 338C is moderately accessible, the other cytidine bases are not accessible.

**FIG 4:**
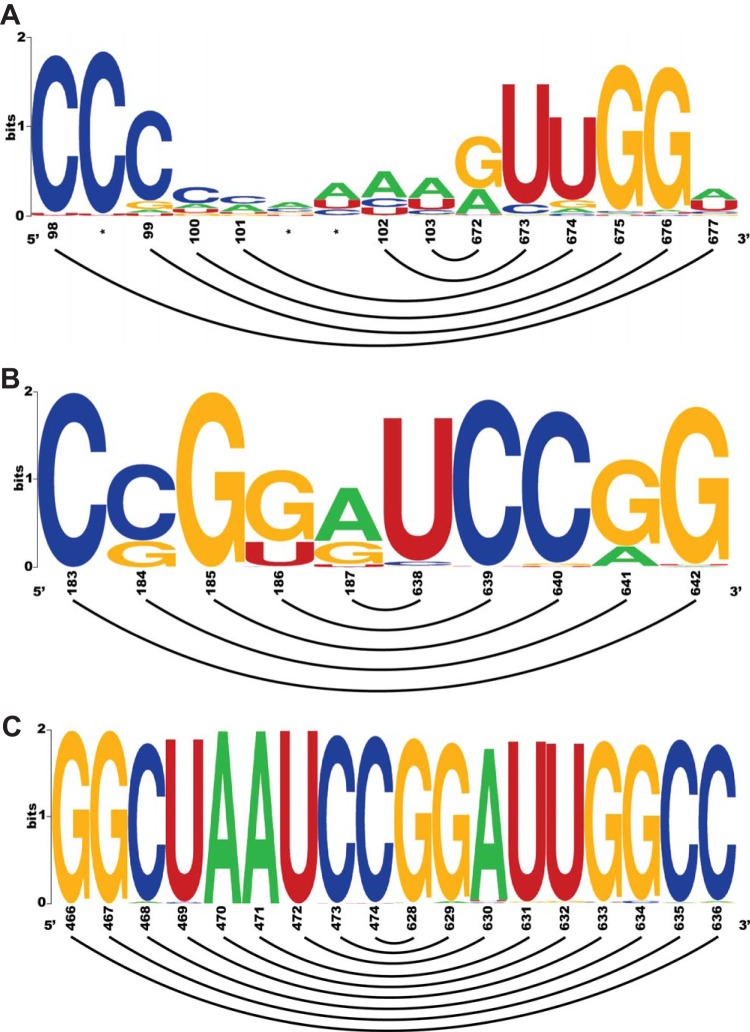
WebLogos showing sequence conservation of positions in proposed long-range interactions LR-1 (A) and LR-2 (B) and in the domain V stem (C). Proposed base-pairing interactions are shown by connecting lines.

Our new model adds roughly 60 residues to the length and substantially rearranges domain V into a complex junction loop. This rearrangement brings a number of functionally important elements into close structural proximity. For example, the pyrimidine-rich region from position 561 to position 581, which plays a role in ribosome recognition (35, 48), was previously thought to bridge domains V and VI but is now fully encompassed within the newly updated domain V. The predicted structure of domain V is highly complex and can be broken into four stem-loop regions that are connected by two multiloops. Sabin vaccine strains of poliovirus have functionally deleterious mutations within this domain. Sabin-like mutations of the CV-B3 5′ UTR have debilitating effects on viral replication and translational efficiency (49). Positions 484A, which is mutated in Sabin 1-like strains, and 485G, which is mutated in Sabin 2-like strains, are both protected from any sort of modification and are involved in Watson-Crick base pairing within a stem near a three-way junction that also branches out into stem-loops 5a and 5b. These two positions exhibit high levels of conservation across our data set of enterovirus A, B, and C species. 484A is conserved in 99% of the sequences, while 485G is present in 97%. The most deleterious is the Sabin 3-like mutation of 473C. In our model, 473C is protected from modification by both SHAPE and base-specific methods and is located near the junction within the basal stem of domain V. Position 473C is conserved across 99% of the enteroviral A, B, and C sequences in our data set. Stem-loop 5d was previously thought to be a discrete domain (domain VI) outside domain V (6). The structure of stem-loop 5d is identical to the domain VI structure of previous models. In our model, stem-loop 5d and the upstream stem-loops share a basal stem (nucleotides 466 to 474 and 628 to 636). This basal stem, which is a newly proposed structural feature of our model, has support from SHAPE analysis and is also supported by base-specific probing. TurboFold II generates this basal stem when constrained by either data set. The sequence of the basal stem is extremely conserved. Of the 18 nucleotides that constitute the basal stem, 14 are protected from SHAPE modification and the other 4 (468C, 469U, 470A, and 471A) are moderately reactive. The 5′ side of the basal stem is highly conserved (Fig. 4) (Stockholm Alignment File [https://unomaha.box.com/s/2osxk3l8xizj3x6usbqd6sqym9qdt8vi]). Positions 466G, 467G, and 472U are perfectly conserved across all of the 1,653 enterovirus A, B, and C sequences analyzed, while 470A and 471A are changed in only one sequence. 469U and 474C are present in 99% of the sequences, and 98% conservation was observed for 468C. Similarly, the 3′ side of the basal stem is highly conserved, with every nucleotide within the region (positions 628 to 636) present in at least 97% of the analyzed sequences. The 469-633 pair has 27 instances of compensatory changes among the 1,653 enterovirus A, B, and C sequences, but there are also 15 examples where a mutation in position 633 does not result in a Watson-Crick pairing. For the other pairs in the basal stem, an average of 0.9% of aligned sequences have mutations that create non-Watson Crick pairs (Stockholm Alignment File [https://unomaha.box.com/s/2osxk3l8xizj3x6usbqd6sqym9qdt8vi]).

The cryptic AUG (positions 591 to 593) within stem-loop 5d is essential for translation, and mutations of this region have been reported to inhibit viral translation (50). Our analysis indicates that this AUG is highly conserved (99%) across enteroviruses A, B, and C species and is mostly inaccessible, except for 591A, which showed moderate reactivity to base-specific probes.

The beginning of domain VI in our proposed model is shifted by about 65 residues from the previously proposed structure due to the increase in the size of domain V in the new model. Our data indicate that domain VI forms a single stem-loop with a single internal loop.

### Long-range interactions.

The secondary-structure model for CV-B3 has two newly identified long-range interactions (Fig. 2, LR-1 and LR-2). LR-1 involves six Watson-Crick base pairs spanning positions 98 to 103 and 672 to 677. Bases 99C, 100C, 673U, 675G, and 676G are not reactive, while other nucleotides constituting the long-range interaction displayed moderate reactivity values. Sequence comparison does not support generalization of this long-range interaction when analyzing enterovirus A, B, and C species (Fig. 4). The 98-to-100 C-rich region is conserved across the aligned enterovirus strains; however, the region also has a large incidence of insertions and deletions. The 672-to-677 region is more conserved. Bases 675G and 676G are present in over 95% of the enterovirus A, B, and C sequences, and over 90% conservation was observed for 673U. 672G and 674U are moderately conserved at 66% and 84%, respectively. Only 7% of the sequences have a guanine at the position corresponding to 677G of the CV-B3 strain 28 5′ UTR. Moreover, among the set of 28 virulent CV-B3 sequences used in TurboFold II to generate the model, the 98-677 pair was conserved in only in 5 of the sequences. Taken together, the data support LR-1 for CV-B3 strain 28, used in our analysis, but the interaction cannot be generalized. We found that LR-1 had sufficient support to be included in our model of CV-B3 because SHAPE probing constraints and base-specific probing constraints independently generated the proposed interaction when analyzed by TurboFold II. However, we cannot say that LR-1 will be a common feature when the structures of other enteroviral 5′ UTRs are determined.

Five Watson-Crick pairs are involved in the long-range binding indicated in box LR-2 (Fig. 2). This interaction is well supported by SHAPE reactivity, and sequence comparison showed a high degree of conservation, together with an example of a pair with compensatory changes (Fig. 4). Nucleotides 640C, 641G, and 642G are moderately reactive to NMIA, while other bases constituting this interaction are not reactive. Bases 183C and 185G were present in over 99% of the enterovirus A, B, and C sequences. 184C and 186G were each found in 74% of the sequences. 187A was 68% conserved and was switched to a guanine in 28% of the analyzed sequences, which maintained the pairing interaction. This was the only pair in either proposed long-range interaction that showed a clear compensatory change. The 638-to-642 region is highly conserved: 638U, 639C, 640C, and 642G were found in over 95% of the sequences. 641G was present in 80% of analyzed enteroviruses. The 184C-641G pair was found in 26 of the 28 virulent CV-B3 sequences, while the other four base pairs were preserved in all of the utilized sequences. Among the enterovirus A, B, and C sequences, 183C-642G and 186G-639C pairs are conserved in over 99% of the strains, while the 185G-640C pair was found in 98% of the sequences.

### An intrinsically disordered RNA region.

**FIG 5:**
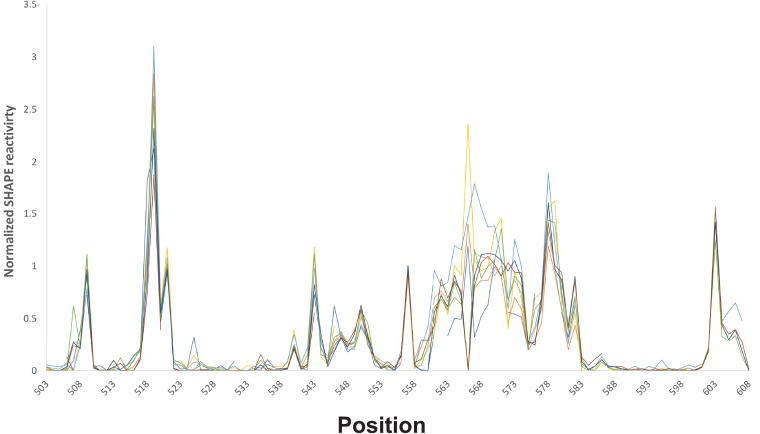
SHAPE reactivity traces by position for positions 503 to 608. Reactivity traces for independent SHAPE experiments are overlaid on the graph. The traces show consistent reactivity for regions upstream and downstream of the proposed intrinsically disordered RNA region (positions 561 to 581).

The pyrimidine-rich single-stranded region leading into stem-loop 5d has been reported to be the ribosomal landing site for the IRES and thus plays an essential role in translation of the viral polyprotein (51). The nucleotides in this single-stranded region (561 to 581) are either highly reactive or moderately reactive, with the exception of 575C, which was not chemically accessible when probed with SHAPE but was moderately accessible to base-specific probes. This pyrimidine-rich region displayed extremely high variability among individual runs when tested with both SHAPE (Fig. 5) and base-specific probes. The modification pattern was highly inconsistent, with bases displaying variable reactivity. As also shown in Fig. 5, the variability in reactivity of the 561-to-581 region was not observed in the regions immediately at the 5′ or 3′ side of this region. Indeed, no other region in the molecule displayed this variability. Positions 561 to 581 represent the longest single-stranded stretch within the CV-B3 5′ UTR. A similar-size single-stranded stretch between positions 223 and 240 was found in an asymmetric internal loop within domain IV. The modification pattern within this single-stranded region was consistent across distinct runs (*n* = 5), suggesting that variability in reactivity to the chemical is not inherent to long single-stranded regions of RNA. Position 566C showed the most variable reactivity in the 5′ UTR but, interestingly, is almost perfectly conserved across enterovirus A, B, and C sequences. Overall, the pyrimidine-rich nature of the entire single-stranded region is highly conserved. However, high rates of insertions and deletions were observed within the 573-to-577 window, and lower conservation levels were observed for positions 570 through 581. The unusual chemical modification behavior of the 561-to-581 region suggests that the positions do not adopt a single, or even small, set of structural states. We propose that this region is intrinsically disordered and represents a feature that functions similarly to intrinsically disordered regions in proteins (36).

## DISCUSSION

The CV-B3 RNA genome is organized into a 742-nucleotide 5′ UTR, a 6,555-nucleotide coding region that produces a 2,185-amino-acid polyprotein, a 98-nucleotide 3′ UTR, and a poly(A) tail (52). This general genome organization is common among enteroviruses and also among all picornaviruses. For all picornaviruses, the highly structured 5′ UTR plays a critical role in gene expression and genome replication. As modeled by the 5′ UTR of CV-B3, replication of enteroviral RNA by the viral RNA-dependent RNA polymerase to produce both negative and positive strands requires a cloverleaf structure on the 5′ end of the genome (9). Beyond the cloverleaf, a large portion of the 5′ UTR functions as an IRES element that is responsible for recruiting ribosomes for translation (26). In enteroviruses, this IRES element is classified as type I. Given the critical functional activities of the 5′ UTR, it is not surprising that many virulence determinants have been localized there (17, 18). Mutagenic, chimeric, and deletion studies have confirmed the close structure-function relationship in the 5′ UTR (17, 49, 53, 54). The prevalence and health consequences of enterovirus infection make improved understanding of the 5′ UTR structure fundamentally important. Recent advances in methods for determining the structure of RNA molecules enable just such improved understanding.

Modern RNA structural analysis represents an amalgamation of biochemistry and computational biology. This is particularly true for chemical probing approaches, where recently introduced chemical agents have joined powerful computational analyses to generate reliable secondary-structure models for large RNAs. High-throughput data-gathering methods, such as capillary electrophoresis and next-generation sequencing, are coupled with custom computational software for quantitative analysis of chemical modification results (55, 56). Folding algorithms have also seen a staggering amount of improvement in both how they predict structures and how they interpret the data from such experiments (57). Minimum-free-energy (MFE) analysis is quite accurate for short RNA regions, but for large RNAs, prediction is much more difficult, even with chemical probing constraints. While MFE analysis remains the most intuitive and popular method of prediction, a new paradigm for structure prediction has been developed. Using base pair probabilities derived from a partition function calculation, a structure with the MEA can be predicted. While MEA structures may not necessarily have the lowest free energy in the ensemble of structures, the pairs in these structures have been shown to be more likely to be correctly predicted than those in their MFE counterparts (58). The accuracy of this approach is further supplemented by the application of probing data in RSample, an algorithm that focuses on the agreement between experimental probing data and estimated probing data by the stochastic sampling of RNA structure models rather than the interpretation of data in the absence of models (59). RSample is the current standard by which SHAPE reactivities are integrated into structure prediction. All of this is combined with comparative sequence analysis in TurboFold II (32) to produce an accurate secondary-structure model.

The structural model we propose for the 5′ UTR of CV-B3 relies upon the results of RSample and TurboFold II, as constrained by our chemical modification values. In some of the proposed double-stranded regions of our model, including some positions in LR-2 and in the basal stem of domain V, there are examples of nucleotides that display moderate or high SHAPE reactivities. It is possible to see reactivity in unexpected places because the SHAPE reactivity value is only one part of the pseudo-free-energy calculation during the structural prediction. As more data accumulate, small adjustments may be necessary to better align the model with experimental results.

A remarkable feature of picornaviral 5′ UTRs is their ability to direct their functional activities independently. Importantly however, despite a well-organized and modular domain structure, these activities require the intact element. Single domains cannot be uniquely ascribed to a function; rather, the domains work in concert (60). Consequently, the structures of the domains and their relationship to each other become important precursors to understanding their functions. In this study, we have defined a new domain organization that directly informs CV-B3 and enteroviral infection efficiency and replication. Among the most influential new features are the long-range interactions, the rearrangement of domain V into a junction loop, and an intrinsically disordered RNA region. Our new model presents a number of targets where mutagenesis is predicted to have deleterious effects on replication.

RNA folding follows a hierarchical pathway in which the primary sequence first forms a base-paired secondary structure that subsequently folds into tertiary modules, which are based primarily on non-Watson-Crick pairing interactions (57). Consequently, a reliable secondary structure is the foundation upon which the RNA structure is built. Our current results provide solid support for a number of previously proposed structural elements and also establish important new elements. The 5′ cloverleaf (domain I), which is so important for replication of the viral genome (24), is confirmed by our probing results. Just downstream of the cloverleaf is a 10-nucleotide C-rich sequence that is largely protected from chemical modification but is not proposed to be part of a base-pairing interaction. This region is recognized by PCBP2 as part of the complex regulating genome replication (41, 61). Protection from modification suggests that these bases adopt a stable structure that restricts backbone flexibility and base accessibility, but the nature of that structure is not currently known. Also, in the connecting region between domains I and II, our model proposes a long-range interaction (LR-1) of 6 bp that involves positions 98 to 103 and 672 to 677. While this interaction is supported by experimental results from virulent CV-B3 strains, it is not supported by a more comprehensive comparison of enteroviral sequences.

In our model, domain II, also called SLII, spans positions 105 to 181. This complex stem-loop contains two bulge loops, a large internal loop, and a capping hairpin loop with a stable stem. Various models for the size and pairing arrangement of domain II have been proposed (6, 20, 28, 38). In our current model, the domain is identical to that in early proposals. A number of studies show that domain II modulates virulence (17, 18, 20, 42, 43). Interestingly, the portion of the domain between positions 118 and 127, where sequences differ the most between virulent and nonvirulent strains, is a long single-stranded region that is protected from modification. As described above for the bases connecting domains I and II, this region must be involved in a higher-order structure that has yet to be identified.

A major rearrangement is proposed for the connecting region between domains II and III, as well as domain III itself. The connecting region contains a long-range interaction (LR-2) involving positions 183 to 187 and 638 to 642. The proposed LR-2 interaction brings the functionally active elements in the 3′ half of the UTR into close proximity with those in the 5′ half. This interaction has the support of one pair with compensatory changes, but most of the bases are highly conserved when enterovirus A, B, and C genomes are compared. Evidence for base pairs is strongest when compensatory changes are present, and conservation of the bases on both sides of a stem is not, by itself, sufficient to prove a secondary structure (62). However, our analysis from TurboFold II combines sequence comparison with SHAPE and thermodynamic constraints. Our model accepted some pairs where SHAPE reactivity was moderately high on one side of the stem and some pairs where sequence comparison did not reveal compensatory base changes. However, the combination of SHAPE constraints, sequence comparison, and MEA values leads to a model consistent with all three lines of evidence. Domain III itself is reduced to a single stem-loop spanning positions 189 to 209. On the 3′ side of the stem-loop, positions 214 to 240, which were previously part of domain III, have been added to a new basal region of domain IV.

Many functional activities are ascribed to domain IV, where host ITAFs, such as PCBP2, and canonical translation initiation factors, such as eIF-4G and eIF-3, have specific interaction sites (48, 63, 64). As in previous proposals, our results show that domain IV has a very long and complex basal stem topped by a junction loop that radiates into three stem loops. Interestingly, nearly all of the functional activities conducted by domain IV map to the junction loop, particularly the C-rich 4a hairpin loop, 4b bulge loop, 4c GNRA tetraloop, and underlying helices (48, 64, 65). Despite its length and complexity, the long stem leading up to the junction loop has not been linked to specific functions. Structurally, the basal extension of the domain 4 stem proposed in our model is intriguing. A long single-stranded region between positions 223 and 240 separates the closing stem from the longer stem beyond. It is unusual for RNA to have such a lengthy unstructured region. However, our SHAPE results support the absence of structure in these positions.

The most significant new insights into enteroviral 5′ UTR structure are found in domain V. These findings are particularly important given the role of domain V in ITAF binding, canonical initiation factor binding, ribosome binding, and virulence (48, 49, 64). Our model for domain V establishes a stable basal helix of nine pairs that defines a large junction loop. The domain encompasses 170 nucleotides and contains three stem-loops that radiate from the central junction. The stem-loop on the 3′ side of the junction includes the bases assigned to domain VI of previous 5′ UTR models (6). The structure of these bases is identical to that of the previously proposed domain VI.

Within large RNA molecules, junction loops provide an architecture that enables conformational and functional flexibility (66). The radiating helices stack coaxially, and the bases in the central core often form tertiary interactions with distant regions (67). As such, they are fundamentally important in functionally active RNAs. The domain V junction loop joins the cloverleaf junction loop as an RNA module with well-established functional importance.

SHAPE analysis of domain V revealed intriguing behavior of the pyrimidine-rich tract between positions 561 and 581. This region has the Y*_n_*-X*_m_*-AUG motif, where Y_n_ is 8 to 10 pyrimidines, X_m_ is 18 to 20 nucleotides, and AUG is a cryptic start codon (33, 34). The Y*_n_*-X*_m_*-AUG region is known to be important for binding multiple factors, including the ribosome, and is present in type I, type II, and type III IRES elements (5, 35). The functional importance of the polypyrimidine region as part of the Y*_n_*-X*_m_*-AUG motif in the proposed IDRR was confirmed by point mutations and deletion mutations shortly after identification of the poliovirus IRES element (47). The same study proposed that the motif represented the ribosome landing pad. Since that time, the Y*_n_*-X*_m_*-AUG motif has been found to be a common feature of type I, II, and III picornavirus IRES elements (60) and has been defined as the binding site for the ribosome, canonical translation factors, and ITAFs (26). The polypyrimidine region has also been modeled as single-stranded region in previous models. Our results show that region 561 to 581 exists as a long single-stranded region in the central core of the domain V junction loop that displays high SHAPE reactivity on average. However, when reactivity is analyzed base by base, there is wild variability between individual runs at every position between 561 and 575, including one position (566) where reactivity fluctuates between highly positive and highly negative SHAPE values. We propose that this region, which includes the Y_n_ segment of the Y*_n_*-X*_m_*-AUG motif, is an IDRR. Intrinsically disordered regions in proteins have been known for some time (68), and they are particularly frequent in proteins that occupy central positions in protein interaction networks, where they bind to multiple partners (69). They are also found in linkers that separate well-structured domains (36). An IDRR in the Y*_n_*-X*_m_*-AUG motif of domain V of the enteroviral 5′ UTR may serve a parallel purpose.

## MATERIALS AND METHODS

### Viral plasmid construction.

A full-length CV-B3 strain 28 (accession no. AY752944.2) genomic construct was engineered and kindly provided by Nora Chapman of the Enterovirus Research Laboratory, University of Nebraska Medical Center, Omaha, NE, USA. The construct was modified to contain a T7 promoter sequence (TAATACGACTCACTATAGGG) upstream of the 5′ UTR and a 38-nucleotide ribozyme sequence (ATGAGGCCGAAAGGCCGAAAACCCGGTATCCCGGGTTC). Upon *in vitro* transcription, the ribozyme sequence self-cleaves to yield the authentic 5′ uridine on the 5′ end of the transcript.

The CV-B3 strain 28 plasmid construct was isolated using a Qiagen (Valencia, CA, USA) QIAprep Spin Miniprep kit in accordance with the manufacturer’s protocol. The isolated plasmid was digested using restriction enzyme Ecl136II to produce the template for *in vitro* transcription that yielded an RNA of 756 bases following ribozyme cleavage.

### *In vitro* viral RNA transcription and purification.

The 5′ UTR was transcribed *in vitro* using a MEGAscript T7 transcription kit (Invitrogen, Carlsbad, CA, USA) according to the protocol provided by the manufacturer. The transcription reaction mixture included 4 μg of the digested template DNA in a total reaction volume of 80 μl. The reaction mixture was incubated at 37°C for 8 h, after which 4 μl of DNase I provided in the kit was added and incubated at 37°C for an additional 30 min to remove the template DNA. The reaction was stopped by adding 460 μl of nuclease-free water and 60 μl of 0.5 M ammonium acetate. The RNA was then purified with phenol-chloroform-isoamyl alcohol (25:24:1), precipitated by incubation with 600 μl of isopropyl alcohol overnight at −20°C, pelleted by centrifugation for 20 min at 4°C, washed with 400 μl of 70% ethanol, and resuspended in 80 μl of 1× TE buffer (5 mM Tris [pH 8.0] and 0.5 mM EDTA), pH 7.6. The RNA was further purified using a MEGAclear transcription clean-up kit (Invitrogen, Carlsbad, CA, USA) according to the manufacturer’s protocol. The purified RNA was stored in small aliquots at −80°C.

### NMIA modification.

NMIA modification reaction mixtures contained 2.4 μg of purified RNA in 60 μl of 0.5× TE buffer, pH 8.0. The RNA was denatured by incubation at 95°C for 3 min and immediately placed on ice. While it was on ice, 30 μl of 3.3× folding buffer (333 mM HEPES, pH 8.0, 20 mM MgCl_2_, 333 mM NaCl) was added to the tube and incubated at 37°C for 20 min to allow the RNA to fold into a stable structure. These buffer conditions and folding conditions match those established and validated during the development of the SHAPE approach (56). Ten microliters of 32.5 mM NMIA solution in dimethyl sulfoxide (DMSO) was added to the RNA and incubated at 37°C for an additional 45 min. The modified RNA was precipitated by incubation with 4 μl of 50 mM EDTA, pH 8.0, 1 μl of 20-μg/μl glycogen, 10 μl of 3 M NaCl, and 300 μl of 100% ethanol at −80°C for 20 min and then pelleted by centrifugation for 20 min at 4°C. The pellet was washed twice with 150 μl of 70% ethanol, dried, and resuspended in 90 μl of 0.5× TE buffer. The control RNA was subjected to all the steps in the protocol except that 10 μl of DMSO was added instead of the NMIA solution.

### DMS modification.

DMS modification reaction mixtures contained 2.4 μg of purified RNA in 100 μl of DMS buffer (40 mM K-cacodylate, pH 7.2, 10 mM MgCl_2_, 50 mM NH_4_Cl). The RNA was denatured by incubation at 80°C for 2 min and slowly cooled to 45°C to allow the RNA to fold into a stable structure. These buffer and folding conditions were chosen to match those established and validated during previous base-specific chemical probing studies (6, 20). Two microliters of 0.4% DMS in 100% ethanol was added to the experimental samples, while 2 μl of DMS buffer was added to the control sample. The samples were then allowed to incubate for 10 min at 37°C. The reaction mixtures were then adjusted to 0.2 μg/μl glycogen, 4 mM EDTA (pH 8.0), and 300 mM NaCl and precipitated with 75% (vol/vol) ethanol. The RNA pellets were dissolved in 90 μl of 0.5× TE and divided into 9-μl (1-pmol) aliquots.

### CMCT modification.

CMCT modification reaction mixtures contained 2.4 μg of purified RNA in 50 μl of CMCT buffer (40 mM K-borate, pH 8.0, 10 mM MgCl_2_, 50 mM NH_4_Cl). The RNA was denatured by incubation at 80°C for 2 min and slowly cooled to 45°C to allow the RNA to fold into a stable structure. These buffer and folding conditions were chosen to match those established and validated during previous base-specific chemical probing studies (6, 20). Fifty microliters of CMCT buffer with or without 6.7 mg/ml CMCT was added and allowed to incubate for 10 min at 37°C. The reaction mixtures were then adjusted to 0.2 μg/μl glycogen, 4 mM EDTA (pH 8.0), and 300 mM NaCl and precipitated with 75% (vol/vol) ethanol. The RNA pellets were dissolved in 90 μl of 0.5× TE and divided into 9-μl (1-pmol) aliquots.

### Primer extension.

The modification sites were analyzed by reverse-transcriptase primer extension. Four primers were utilized to cover the entire 5′ UTR. The sequences and priming positions of the primers are provided in Table 1.

**TABLE 1:** Primers for primer extension of CV-B3 strain 28 5′ UTR RNA

Primer extension starting position	Sequence (5′–3′)
168	GGTAACAGAAGTGCTTGATC
368	CCCCATGGGCAGGCCGCC
627	AGTCACCGGATGGCCAATCC
722	TTGCTGTATTCAACTTAAC

Two sets of the four oligonucleotide primers were designed: one set of primers was labeled on the 5′ end using VIC fluorescent dye, while the other set of primers was labeled with 6-carboxyfluorescein (6-FAM) fluorescent dye. The primer extension protocol was adopted from that of Burrill and Andino (70). Two fluorescently 5′-labeled versions (6-FAM and VIC) of each primer were used in primer extension. The extension reaction mixtures included experimental (plus) and control (minus) RNAs, as well as dideoxy-sequencing reaction mixtures needed for ShapeFinder analysis (see below). For each experiment, eight primer extension reactions were performed: 1 plus, 2 minus, and 3 to 8 for sequencing. For each run, 9 μl (1 pmol) of RNA was denatured at 95°C for 3 min and placed on ice. Two microliters of 1 μM VIC-labeled primer was added to reaction mixtures 1 through 4, and the same amount of 6-FAM-labeled primer was added to reaction mixtures 5 through 8. The reaction mixtures were incubated at 65°C for 5 min and at 35°C for 10 min and placed on ice. Nine microliters of the appropriate RT mixture was added. The basic RT mixture (1 μl of SuperScript III [Invitrogen], 4 μl of 5× first-strand buffer [Invitrogen], 1 μl of 0.1 M dithiothreitol, 1 μl of the 10 mM mixture of deoxynucleoside triphosphates, and 2 μl of water) was added to reaction mixtures 1 and 2. The basic mixture was supplemented with 1 μl of 10 mM ddATP for reaction mixtures 3 and 5 through 8 and 1 μl of 10 mM ddCTP for reaction mixture 4. The reaction mixtures were incubated at 52°C for 15 min and placed on ice. To degrade the RNA template, 2.5 μl of 1 M NaOH was added to each reaction mixture, and the mixtures were incubated at 98°C for 15 min and then placed on ice. To neutralize the reaction, 2.5 μl of 1 M HCl was added. The VIC and 6-FAM reaction mixtures were pooled in a pairwise manner (1 and 5, 2 and 6, 3 and 7, and 4 and 8) and diluted 2-fold with water. The four pooled reaction mixtures were adjusted to 0.2 μg/μl glycogen and 300 mM sodium acetate (NaOAc) (pH 5.2) and precipitated by adding ethanol to 75% (vol/vol). The cDNA pellets were washed twice with 75% ethanol and dissolved in 10 μl of HiDi formamide (Life Technologies). Capillary electrophoresis was performed by the University of Nebraska Medical Center (UNMC) sequencing core facility according to their fragment analysis protocol. Each of the four pooled reaction mixtures was analyzed in a separate capillary during capillary electrophoresis.

### Quantitative analysis of reactivities.

Analysis of raw electropherograms, which contained data for fluorescence intensity versus elution time, was carried out as described previously (70). The data files were analyzed in ShapeFinder (37). Data from the 6-FAM and VIC channels for each of the four capillaries were first combined into a single file. A fitted baseline adjustment with a window width of 200 was applied to all eight channels. Cubic mobility shifts were performed manually on the four 6-FAM channels to align these identical ddATP sequencing reactions. The shift values from these alignments were then applied to the appropriate VIC channels, aligning the experimental (plus), control (minus), and sequencing channels with one another. The data from the four 6-FAM channels were then removed before continuing the analysis. Signal decay correction was applied to each of the four remaining VIC channels. The region of interest for the signal decay correction and further analysis was determined by visually inspecting the quality of peaks within the plus channel, and the same window was applied to all four channels. The rescale factor and equation parameters (*A*, *q*, and *C*) were kept at their default values of 10,000, 1,000,000, 0.999, and 10,000, respectively. A scale factor was then applied to the minus channel so that most peaks were of a height equal to or less than that of the corresponding plus peak. This factor was typically between 0.3 and 0.7. Alignment and integration were then performed over a range equal to or narrower than that used for signal decay correction. After manual inspection and correction of peaks, the ShapeFinder software performed a whole-channel Gaussian integration to quantify all individual peak areas, and the minus peak areas were subtracted from the plus areas. The raw reactivity data for each primer were normalized using a 2% to 8% normalization scheme (56). Accordingly, the top 2% of data were taken as outliers. The average of the next 8% of the data was found, and each datum, including the top 2%, was then divided by this average reactivity. At least three independent runs were completed for each primer. The normalized reactivity values for each 5′ UTR position were tested for outliers using Dixon’s Q test at a 95% confidence interval. The outliers were excluded from further analysis.

### RNA structural prediction.

The RNA secondary structure was modeled with TurboFold II, a computational tool that incorporates experimental probing data with sequence comparison to inform predictions of RNA secondary structure (32). For the sequence comparison data set, genomes from 28 strains of coxsackievirus experimentally shown to be virulent and also present as full-length genomes in the database were selected. CV-B3 strain 28 was included in this data set. The first 700 nucleotides of these genomes were input into the TurboFold II analysis. SHAPE data were applied to CV-B3 strain 28 during the prediction, with RSample enabled using its default parameters (59). The SHAPE slope and intercept were changed to 2.6 and −0.8, respectively (56). The prediction was performed in MEA mode with default settings (58). The resultant connectivity table (CT) files were converted into image files with the draw algorithm from RNAstructure (71).

### Multiple-sequence alignment.

Analysis of sequence conservation for enterovirus 5′ UTRs was performed by multiple-sequence alignment in Clustal Omega using the default parameters (72). Complete full-length genomes of 1,653 viral sequences from the enterovirus A, B, and C species were obtained from the NCBI database (see Table S3 in the supplemental material). The CV-B3 strain 28 sequence (accession no. AY752944.2) was set as the reference. Sequences selected for the data set had to be greater than 5,000 nucleotides and had to be full length to the 5′ end. These sequences were trimmed to display the 5′-most 700 nucleotides and were loaded into Clustal Omega as a single FASTA file. The sequence alignment is provided as a Stockholm Alignment File (https://unomaha.box.com/s/2osxk3l8xizj3x6usbqd6sqym9qdt8vi).

## Supplementary Material

Supplemental file 1

Supplemental file 2

Supplemental file 3
